# First metatarsophalangeal joint arthrodesis versus proximal phalanx hemiarthroplasty for hallux rigidus: feasibility study for a randomised controlled trial

**DOI:** 10.1186/1745-6215-15-79

**Published:** 2014-03-13

**Authors:** Hiren Maganlal Divecha, Aamir I Zubairy, James L Barrie, Shivashanker Aithal, Benjamin Fischer, Thomas Fanshawe, Asim Rajpura

**Affiliations:** 1 East Lancashire Hospitals NHS Trust, Royal Blackburn Hospital, Haslingden Road, Blackburn BB2 3HH, UK; 2 Department of Primary Care Health Sciences, University of Oxford, Radcliffe Observatory Quarter, Woodstock Road, Oxford OX2 6GG, UK

**Keywords:** Hallux rigidus, Proximal phalanx hemiarthroplasty, Arthrodesis, Metatarsophalangeal joint

## Abstract

**Background:**

Osteoarthritis of the first metatarsophalangeal joint (hallux rigidus) leads to pain and poor function and mobility. Arthrodesis is the gold standard treatment for end-stage disease. Total joint arthroplasties have been attempted, but early loosening has been attributed to dorsally directed shear forces on the metatarsal component. Metallic proximal phalangeal hemiarthroplasty theoretically avoids this. Whilst early results are promising, no comparative trials exist comparing this to arthrodesis.

**Methods/Design:**

The primary objectives are to determine the range of outcome scores between the two treatment arms (to inform a power calculation). Outcome measures will include the MOXFQ, AOFAS-Hallux and EuroQol EQ-5D-5 L. Secondary objectives are to determine the accrual rate, dropout rate and trial acceptability to both patients and surgeons. These data will allow the development of a larger trial with longer follow-up.

This is a prospective randomised controlled single-centre study comparing proximal phalanx hemiarthroplasty (AnaToemic, Arthrex Ltd., Sheffield, UK) with arthrodesis (15 patients in each arm). Randomisation will be performed using a 1:1 allocation ratio in blocks of six.

Patients meeting the eligibility criteria will be recruited from three foot and ankle consultant surgeon’s clinics (East Lancashire Hospitals NHS Trust). If agreeable, informed consent will be obtained before patients are randomised.

The outcome measure scores will be completed pre-operatively and repeated at 6 weeks, 3 months and 12 months. A radiological review will be performed at 6 weeks and 12 months to determine rates of loosening (hemiarthroplasty) and union (arthrodesis). Data on length of stay, return to work, complications and re-operation rates will also be collected.

The analysis will compare the change in outcome scores between treatment groups at all follow-up time points. Scores will be compared using a Student *t*-test, adjusting for scores at baseline.

This study will be conducted in accordance with the current revision of the Declaration of Helsinki (1996) and the ICH-GCP Guideline (International Conference on Harmonisation, Good Clinical Practice, E6(R1), 1996). This study has been approved by the sponsor, the Trust Research & Development office. Ethical approval has been received from the National Research Ethics Service (North East: 12/NE/0385 for protocol version 5.3 dated 3 June 2013).

**Trial registration:**

Current Controlled Trials ISRCTN88273654

## Background

First described in 1887, hallux rigidus refers to the symptoms of degenerative arthritis of the first metatarsophalangeal joint (MTPJ) [1]. It is characterised by pain, loss of motion, primarily dorsiflexion and periarticular osteophyte formation. More common in females, hallux rigidus is thought to affect 1 in 45 individuals over the age of 60 years [2]. Trauma is a frequently cited aetiological factor, especially for unilateral hallux rigidus [3]. Generalised osteoarthritis and inflammatory arthropathies, such as rheumatoid arthritis, can also result in hallux rigidus. Irrespective of the causative factors involved, end-stage disease results in articular cartilage loss and loss of joint space. Subsequent pain and loss of motion results in abnormal gait patterns and can interfere with simple daily tasks such as walking and stair climbing [4]. The severity of hallux rigidus can be classified using the system suggested by Coughlin and Shurnas [5]. Early disease, grades 0 to 2, can respond well to non-operative measures such as non-steroidal anti-inflammatory medication and footwear modification. Cheilectomy to remove impinging dorsal osteophytes can also be successful in early cases as the disease initially affects the dorsal portion of the joint. End-stage disease, grade 3 to 4, does not, however, respond as well to such measures and remaining options include joint arthrodesis or arthroplasty [6].

First MTPJ arthrodesis is currently considered to be the gold standard for the treatment of end-stage hallux rigidus, with reported union rates ranging between 90% and 100% [7]. Arthrodesis does, however, predispose the patient to interphalangeal joint osteoarthritis and the loss of dorsiflexion can interfere with activities such as kneeling and squatting, and wearing footwear with raised heels can be problematic [8,9]. Post-operative rehabilitation can also be prolonged and there is up to a 10% risk of non-union, which may require a revision procedure [7]. Thus as an alternative, arthroplasty of the first MTPJ has been attempted.

Silicone implants were initially used following their successful implantation in the hand. Early results were promising with high rates of patient satisfaction [10]. Unfortunately, problems with silicone wear, osteolysis and foreign body reactions limited their longevity [11,12]. Silicone has therefore been suggested to be a material not suitable for first MTPJ replacement due to its poor ability to withstand the forces encountered.

This led to the development of metallic implants in both total toe replacement and hemiarthroplasty designs [13]. Total toe implants have demonstrated good early patient satisfaction [14]. Early loosening, however, was once again a problem [14,15]. This is thought to be due to the large dorsally directed shear forces the metatarsal implant is exposed to during toe-off [16].

Metallic proximal phalangeal hemiarthroplasty (PPH) may therefore avoid both problems related to silicone wear and loosening related to the shear forces exerted on metatarsal head implants. The literature on the results of these implants is, however, sparse and somewhat conflicting. Townley and Taranow [17] published the largest series of 279 PPHs with follow-up ranging from 10 months to 33 years. This retrospective review reported good or excellent results in 95% of subjects. More recently Sorbie and Saunders [18] published their series of 23 patients treated with PPH with follow-up ranging from 34 to 72 months. They report an improvement in AOFAS score from 57 to 88 with no cases of loosening or osteolysis. In contrast Raikin *et al*. [19] found inferior results for PPH compared to arthrodesis. They conducted a retrospective review of 48 feet, 27 with arthrodeses and 21 with PPHs. Of the arthrodesis group, 85% were deemed to have had a good outcome based upon patient-reported satisfaction, versus 60% in the PPH group, with 5 (24%) of the PPH group requiring re-operation due to loosening. They concluded arthrodesis is a more predictable technique for dealing with hallux rigidus.

The literature is therefore divided regarding the efficacy of PPH for the treatment of hallux rigidus. Studies to date have all been retrospective in nature. The only prospective randomised trial so far has compared total joint arthroplasty versus arthrodesis, and concluded outcomes for arthrodesis were better than arthroplasty [20]. A similar prospective randomised study therefore is required to compare outcomes of PPH versus joint arthrodesis accurately. We therefore propose a randomised controlled trial comparing the AnaToemic PPH (Arthrex Ltd., Sheffield, UK) to first MTPJ arthrodesis. This prosthesis shares similar design features to the BioPro First MPJ Hemi Implant (BioPro, Michigan, USA) but has a better cost profile. Early results reported by Kissler and colleagues are promising, with good improvements in pain scores and revision rates comparable to non-union rates observed with arthrodesis [21].

## Methods/Design

This study will run as a prospective single-centre randomised controlled trial with two parallel treatment arms containing 15 participants each and comparing PPH with arthrodesis (the current gold standard for the treatment for hallux rigidus). The primary objective is to determine the range of improvement in outcome scores following these procedures. The secondary objective is to determine the patient recruitment rate, trial dropout rate (including those lost to follow-up) and the acceptability of the trial as a process to surgeons and patients (by way of qualitative interviews). The combination of the primary and secondary objectives will be used in a power calculation and to design a larger, multi-centre randomised, controlled trial with longer follow-up.

### Patient recruitment

Patients will be screened and recruited by three consultant foot and ankle orthopaedic surgeons from outpatient clinics (in the East Lancashire Hospitals NHS Trust) after confirmation of diagnosis and eligibility (see Table 1). Screening will continue until the target sample size is reached (15 patients in each arm). The number of patients to be recruited has been based on discussions with the trial statistician. There is no published data using the MOXFQ tool to assess differences between PPH and arthrodesis. Therefore, a power calculation was not performed.

**Table 1:** Eligibility criteria

**Inclusion**	**Exclusion**
1. Coughlin grade 3 to 4 hallux rigidus with near constant pain and stiffness at least at extremes of passive motion +/− mid range of motion pain, where conservative treatment measures have failed	Inflammatory arthritis
	Active infection
	Severe vascular or neurological deficit affecting the lower limbs
	Hallux valgus angle >15°
	Inadequate or poor quality bone stock
2. Aged 45 years or above	Any previous surgery to the first MTPJ other than a cheilectomy
3. Male or female	
4. Able to give informed consent	
	Significant and symptomatic interphalangeal joint or sesamoid-metatarsal osteoarthritis

Patients who meet the eligibility criteria will be provided with a patient information sheet and given 1 to 2 weeks to go over this information. If agreeable, informed consent for entry into the trial will be obtained from patients before randomisation is performed. Randomisation will be performed using a random computer generated sequence with a 1:1 allocation ratio in blocks of six. The online website Sealed Envelope [22] has been used to do this. Allocation concealment will be ensured by using a locked spreadsheet, which will only reveal the allocated treatment arm after a patient’s hospital number and study number have been entered. Given the nature of surgical procedures, the only person involved in this trial who will remain blinded to treatment arm allocation is the statistician. Surgeons and patients cannot be blinded to the treatment arm. The outcome measures used in this study are all patient administered and therefore this study does not require a blinded assessor.

### Interventions

Surgery will be performed by one of the three consultant orthopaedic foot and ankle surgeons who are FRCS (Trauma & Orthopaedics) qualified and on the General Medical Council’s Specialist Register.

#### Arthrodesis arm

The procedure will be carried out under either general or regional anaesthesia as directed by the anaesthetist and patient. A local anaesthetic ankle block to the tibial, saphenous, deep and superficial peroneal nerves will be administered prior to the start of the procedure using 20 ml 0.5% bupivicaine. The first MTPJ will be exposed through a longitudinal dorsal or medial skin and capsular incision and the joint surfaces will be prepared, arthrodesed in 10° of dorsiflexion and held with two crossed headless compression screws. Post-operatively the patients will be mobilised in a wedge shoe for 6 weeks.

#### Hemiarthroplasty arm

The hemiarthroplasty device used will be the AnaToemic PPH (Arthrex Ltd., Sheffield, UK) The same surgical approach as used for the arthrodesis group will be employed. Adhesions between the sesamoids and the metatarsal head will be released and osteophytes excised. The component will be inserted uncemented as per the manufacturer’s guidelines, including carrying out a cheilectomy if required to ensure a minimum arc of motion of 80° is achieved. Active and passive mobilisation will be commenced on the second day post-operatively, and a wedge shoe will be worn for 6 weeks. Following this period, these patients will have physiotherapy aimed at increasing the range of motion of the joint.

### Data collection

The primary outcome measure, the MOXFQ, was chosen as it is a validated, patient-reported, foot and ankle questionnaire. It comprises 16 questions and is represented by three domains (walking/standing, pain and social interaction). The MOXFQ has been shown to be reliable, valid, responsive and acceptable in validation studies [23,24].

The AOFAS-Hallux score comprises three areas: pain, function and alignment. This is a clinician-administered questionnaire, scored out of 100. The question relating to MTPJ range of motion will be removed for all patients in both treatment arms post-operatively. This is because the arthrodesis treatment arm will automatically score 0 for this (an arthrodesis by definition is a fused joint). Therefore, post-operative AOFAS-Hallux scores will be scored out of 90. The AOFAS score is very popular for reporting foot and ankle outcomes. However, it does not have extensive or rigorous supporting validation studies. Baumhauer *et al*. [25] raised concerns regarding this score’s reliability and validity in the activity subscale. Ibrahim *et al*. [26] reported satisfactory reliability and responsiveness with acceptable validity at best. For this reason, we have opted to use the MOXFQ score as a primary outcome measure.

The EuroQol EQ-5D-5 L is a standardised measure of health status [27]. It comprises five dimensions (anxiety/depression, mobility, self-care, usual activities and pain/discomfort) and each dimension has five levels of patient-reported severity. When referenced to population-standardised datasets, this provides a health status index value. A visual analogue score for perceived health state makes up the second part of the EQ-5D-5 L.

Trial data will be collected using a structured data collection form, which will include basic patient demographic details and all relevant study questionnaires. These will be completed in paper format. All patient trial paperwork will be stored in individual files in a secure, locked cabinet in the Research & Development main office. Data fields will be checked for completeness and entered into a secure password-protected spreadsheet. This will be stored on a Trust approved, encrypted USB stick and kept locked along with the Trial Site File and patient trial folders.

For all patients, basic demographic data will be recorded following trial consent and randomisation. Outcome scores (MOXFQ, AOFAS and EQ-5D-5 L) will be completed preoperatively and then post-operatively (see Table 2). Details regarding length of hospital stay, return to work, complications and any episodes of repeat surgery will also be collected.

**Table 2:** Schedule of screening, enrolment, randomisation, intervention and assessment

	**Study period**						
	**Screening**	**Enrolment and randomisation**	**Surgery**	**Post-surgery**			
Time point (weeks)	0	2	6 to 12	2	6	12	56
Enrolment:							
Eligibility screen	X						
Informed consent		X					
Randomisation		X					
Intervention:							
Arthrodesis			X				
Hemiarthroplasty			X				
Assessment:							
Patient demographics		X					
MOXFQ		X			X	X	X
AOFAS-Hallux		X			X	X	X
EQ-5D-5 L		X			X	X	X
Clinical review				X	X		X
Radiological review					X		X

At the 12-month follow-up, patients will additionally have a short structured interview with the research nurse to ascertain the acceptability of the trial process as a whole, the acceptability of the randomisation process and any aspects that could be improved. The operating surgeons (both senior authors) will also undergo similar structured interviews at the conclusion of the trial. This qualitative data will be collated and used to determine what areas of the trial design and conduct need to be addressed before embarking on a larger prospective multi-centre randomised control trial.

### Statistical methods

The primary analysis will compare MOXFQ scores between the two treatment groups at 12 months using a *t*-test, adjusting for the score at baseline. A similar analysis will be carried out to investigate differences in secondary outcomes (AOFAS-Hallux and EQ-5D-5 L) between the two treatment groups. Effect sizes will be presented as a point estimate, 95% confidence intervals and *P* values. Rates of patient dropout will be reported at each time point. The observed effect size will be used in a power calculation for a future definitive trial.

### Ethics and dissemination

This study will be conducted in accordance with the current revision of the Declaration of Helsinki (1996) and the ICH-GCP Guideline (International Conference on Harmonisation, Good Clinical Practice, E6(R1), 1996). The research protocol, patient information sheets and trial consent forms have been reviewed and approved by the sponsor, the Trust Research & Development office. Ethical approval has been received from the National Research Ethics Service (North East: 12/NE/0385 for protocol version 5.3 dated 3 June 2013). The trial has been registered as a controlled trial (ISRCTN: 88273654) [28].

We aim to publish one main trial outcome paper from this study and present the results at appropriate national and international orthopaedic conferences. The data from this study will hopefully provide a range of changes in outcome measures, which will then allow a power calculation. Together with data collected on patient accrual rate, dropout rate and trial acceptability (to patients), we aim to develop a larger prospective randomised controlled trial (possibly multi-centre based) with longer follow-up.

### Trial status

The trial is currently recruiting participants.

## Abbreviations

MTPJ: metatarsophalangeal joint; PPH: proximal phalangeal hemiarthroplasty.

## Competing interests

The authors declare that they have no competing interests.

## Authors’ contributions

HD and AR help to conceive and design the study, helped to write the manuscript (draft and critical revisions) and gave final approval for the manuscript. AIZ and JLB helped to conceive and design the study, helped to write the manuscript (critical revisions), gave final approval for the manuscript and are grant holders. SA helped to conceive and design the study, helped to write the manuscript (critical revisions) and gave final approval for the manuscript. BF helped to design the study and write the manuscript (draft and critical revisions) and gave final approval for the manuscript. TF helped to design the study, provided statistical expertise, helped to write the manuscript (draft) and gave final approval for the manuscript.
